# Effect of Influenza Vaccination on Mortality and Heart Failure Hospitalization in Heart Failure Patients

**DOI:** 10.3390/vaccines13101055

**Published:** 2025-10-15

**Authors:** Chatpetch Maneesopit, Arintaya Phrommintikul, Jackrapong Bruminhent, Ammarin Thakkinstian, Teerapat Yingchoncharoen

**Affiliations:** 1 Faculty of Medicine Ramathibodi Hospital, Mahidol University, Bangkok 10400, Thailand; only.petch@gmail.com (C.M.); jackrapong.brm@mahidol.ac.th (J.B.); ammarin.tha@mahidol.ac.th (A.T.); 2 Department of Medicine, Faculty of Medicine, Chiang Mai University, Chiang Mai 50200, Thailand; arintaya.p@cmu.ac.th

**Keywords:** influenza vaccine, heart failure, mortality

## Abstract

Background: The association of influenza vaccination and heart failure (HF) hospitalization and mortality in Thai HF patients is unknown. Objective: We wished to investigate associations between receiving an influenza vaccine and all-cause death or HF hospitalization in HF patients. Methods: We retrospectively reviewed medical records from two large tertiary-care centers in Thailand (Ramathibodi Hospital and Maharaj Nakorn Chiang Mai Hospital) with newly diagnosed heart failure between 2013 and 2020 in an outpatient clinic. We examined the relationship between influenza vaccination and outcomes in a propensity-adjusted model. Results: Of 588 patients, 181 (31%) received an influenza vaccination. During a median follow-up of 57 months, influenza vaccination was associated with a 56% reduction in the risk of HF hospitalization or death (HF hospitalization or all-cause death: HR: 0.44; 95% CI: 0.31–0.63; *p* < 0.001) in an unadjusted analysis. After propensity score adjustment, influenza vaccination, however, was not associated with a reduced risk of all-cause death but was associated with a reduced risk of HF hospitalization (ATE: 3.06 years; 95% CI: 0.14 to 5.98; *p* = 0.04). Conclusions: In patients with HF, influenza vaccination was not associated with a reduced risk of the composite of all-cause death or HF hospitalization after adjustment for confounders.

## 1. Introduction

Cardiovascular diseases (CVDs) remain the leading cause of morbidity and mortality worldwide, with heart failure (HF) representing one of the most burdensome conditions both in terms of clinical outcomes and healthcare expenditure. Patients with HF experience frequent hospitalizations, impaired quality of life, and elevated mortality rates despite advances in guideline-directed medical therapy.

In patients with heart failure, respiratory tract infections, including influenza infections, are one of the most important triggers for acute HF exacerbation and are associated with high in-hospital mortality [1]. HF patients with concomitant influenza infection also have a higher risk of acute respiratory failure, acute kidney injury, and prolonged hospital stays [2].

Numerous randomized control trials (RCTs) and cohort studies have been conducted to assess the effects of influenza vaccinations on all-cause and cardiovascular (CV) mortality and HF hospitalization in HF patients [3,4,5]. A meta-analysis of six RCTs involving mixed myocardial infarction (MI) and HF patients showed that an influenza vaccine could lower the risk of all-cause mortality by approximately 28% (HR = 0.72, 95% CI: 0.54–0.95) and the risk of CV mortality by 37% (HR = 0.63, 95% CI: 0.42–0.95) [6,7,8,9,10]. However, evidence from meta-analyses of cohort studies carries a high risk of bias and low certainty.

These benefits were consistent in meta-analyses from six to eight cohort studies, which showed a 20–30% reduction in all-cause mortality regardless of influenza seasonality but no significant effect on CV mortality or all-cause hospitalization [10]. However, observational evidence from cohort studies carries a high risk of bias and low certainty.

To address these limitations, some clinical trials were conducted. Secondary analyses of RCTs using propensity score analysis have yielded conflict findings: the PARADIGM-HF trial found a 20% reduction in all-cause mortality with influenza vaccination while the PARALLEL-HF trial found no significant effect. Interpretation is limited by the low vaccination rate among Asian participants in PARADIGM-HF and the small sample size (*n* = 223) in PARALLEL-HF [4,11].

However, a few limitations of these two studies should be addressed: the PARADIGM-HF study included HF patients from US, Europe, and Asia, in which only a low percentage of influenza vaccines was performed in Asians; meanwhile, the PARALLEL-HF trial had very small sample size of 223.

Three other emulated RCTs from cohort data applying propensity score matching found substantial reductions in all-cause mortality from about 41% to as high as 96% in Spanish studies and 20% in an Israeli study. Nonetheless, verification of the real-world effectiveness of the influenza vaccine in Asian populations remains scarce [12,13,14].

Annual influenza vaccination is widely recommended for patients with chronic cardiovascular conditions, including HF, as an evidence-based preventive strategy. Professional societies such as the American Heart Association, the American College of Cardiology, and the European Society of Cardiology endorse influenza vaccination as part of comprehensive care in HF patients. Similarly, the World Health Organization has prioritized individuals with chronic heart disease for annual influenza vaccination campaigns. Despite these recommendations, the actual uptake of influenza vaccination among patients with HF remains suboptimal worldwide. A variety of factors contribute to this gap, including limited awareness among patients, inconsistent emphasis by healthcare providers, vaccine hesitancy, and challenges in healthcare system delivery.

Unlike countries in temperate climates, where influenza follows a clear seasonal pattern, Thailand experiences year-round transmission without marked seasonality [6]. This epidemiological difference raises the hypothesis that influenza vaccination could improve prognosis and reduce HF-related hospitalizations in Thai patients, potentially offering an even greater relative benefit than that in countries with seasonal influenza peaks.

In Thailand, seasonal influenza vaccination has been progressively integrated into the national immunization strategy over the past two decades. Since 2008, the Ministry of Public Health has provided influenza vaccines free of charge for prioritized groups, including older adults (≥65 years), pregnant women, young children, healthcare personnel, and individuals with chronic diseases. Despite these efforts, overall coverage remains modest, with uptake rates among elderly and high-risk populations typically ranging from 10 to 20% in most years, substantially lower than in high-income countries. Importantly, vaccination in Thailand is not mandatory but strongly recommended, and access outside the national program often requires out-of-pocket payment. Implementation challenges—such as limited supply, distribution constraints, and variable public awareness—have contributed to suboptimal vaccine uptake. These features highlight the need for locally generated evidence on the cardiovascular benefits of influenza vaccination, particularly in patients with heart failure, to support health policy and increase coverage in this vulnerable population [15,16,17].

Given these gaps, we aimed to examine the association between influenza vaccination and adverse clinical outcomes in a cohort of HF patients treated at a tertiary-care hospital in Thailand. Specifically, we investigated whether influenza vaccination was associated with reduced risks of all-cause mortality and HF hospitalization. By providing data from a tropical, middle-income setting, our study seeks to complement existing international evidence and contribute to a more comprehensive understanding of the role of influenza vaccination in the management of HF.

## 2. Methods

### 2.1. Study Design and Patient Selection

We conducted a retrospective non-matched cohort study at two academic medical centers in Thailand: Ramathibodi Hospital, Bangkok, and Maharaj Nakorn Chiang Mai Hospital, Chiang Mai. The enrollment period was from 1 January 2013 to 1 December 2020, during which all eligible patients with a new diagnosis of HF were identified. Outcomes were assessed through hospital medical records, and patients were followed until 30 June 2021, ensuring that those diagnosed near the end of the enrollment period had at least six months of follow-up.

Adult patients (aged ≥18 years) who were newly diagnosed with heart failure (HF) in internal medicine clinics during this period were identified using International Classification of Diseases, Tenth Revision (ICD-10) codes for HF (I50, I500, I509), cardiomyopathy (I42), and ischemic cardiomyopathy (I255). Medical records were reviewed by internist physicians to confirm the diagnosis of HF. Confirmation required documentation of clinical symptoms and signs consistent with HF, supported by echocardiographic findings or physician assessment in accordance with contemporary guidelines.

Patients were eligible for inclusion if they had a new diagnosis of HF during the study period and complete medical records allowing for the assessment of baseline demographics, comorbidities, medications, and outcomes. Patients were excluded if they were lost to follow-up for more than one year after the index diagnosis, as outcomes could not be reliably assessed in these cases (*n* = 23). All patients were newly diagnosed with HF during the study period; therefore, no prior HF cases were included. Vaccination status did not determine eligibility for enrollment, and all patients meeting the inclusion criteria were followed regardless of their subsequent vaccination status.

Attrition occurred during the patient selection process. Specifically, 23 patients were excluded because they were lost to follow-up for more than one year, and 203 were excluded due to incomplete or unavailable data. These exclusions were applied prior to analysis, resulting in a final analytic cohort of 588 patients. No further attrition occurred during follow-up, as outcomes were ascertained through hospital electronic medical records.

Data were extracted from electronic medical records, including demographics (age, sex, body weight, height, body mass index [BMI], and blood pressure), comorbidities (hypertension, diabetes mellitus, dyslipidemia, chronic kidney disease, and etiology of HF), echocardiographic parameters such as left ventricular ejection fraction (LVEF), and laboratory results. Information on prescribed medications was collected, including angiotensin-converting enzyme inhibitors (ACEIs), angiotensin receptor blockers (ARBs), angiotensin receptor–neprilysin inhibitors (ARNIs), beta-blockers, mineralocorticoid receptor antagonists (MRAs), statins, and antiplatelet agents. Medication use was defined as at least one filled prescription within 6 months before or 30 days after the HF diagnosis. The MAGGIC heart failure score was calculated for each patient using available clinical and laboratory data.

### 2.2. Influenza Vaccination Status

Influenza vaccination status was determined through longitudinal review of the hospital electronic medical records (EMRs) at both study sites. Vaccination entries were routinely documented by healthcare providers at the time of administration and were available for all subsequent clinical visits. Patients were considered vaccinated if they had received at least one influenza vaccine after the diagnosis of HF and before the end of follow-up (30 June 2021). Influenza vaccination status was determined from the hospital electronic medical record (EMR) vaccination registry, which is based on provider-entered documentation at the time of vaccine administration. In our setting, influenza vaccination is typically administered during outpatient visits, with occasional inpatient administration. To ensure data accuracy, all enrolled patients (or their caregivers) were contacted by telephone to confirm the dates and history of influenza vaccination. These confirmations verified that no participants had received an influenza vaccination prior to the index heart failure (HF) diagnosis. Vaccinations performed outside the study hospitals, such as in primary care or community clinics, were not captured due to the absence of a centralized national vaccination database in Thailand. Thus, the term any follow-up period refers to the entire observation window after HF diagnosis until 30 June 2021. For each vaccinated individual, the month of vaccination and the cumulative number of doses during the study period were recorded. Because the inclusion criteria required a new HF diagnosis during the enrollment period, no patients had received an influenza vaccination before the index HF diagnosis. Importantly, vaccination status did not determine study eligibility; all patients with newly diagnosed HF during the enrollment period were included, regardless of subsequent vaccination status.

### 2.3. Outcomes

The primary endpoint was a composite of all-cause mortality or HF hospitalization. Secondary endpoints included each component of the composite outcome as well as cardiovascular (CV) mortality. For patients who experienced more than one HF hospitalization during the study period, only the first hospitalization after the index diagnosis was included in the primary time-to-event analysis. This ensured that each patient contributed only one event to the analysis, and recurrent hospitalizations were not considered as separate outcomes.

HF hospitalization was defined as admission with clinical signs or symptoms of decompensated HF requiring intravenous loop diuretic therapy (e.g., furosemide) in either the emergency department or an inpatient ward for >24 h.

CV mortality was defined as death attributed to acute coronary syndrome (ACS), HF, or stroke, as documented in the medical record and verified by independent physician review. Outcome data were obtained from hospital medical records, and the follow-up for outcomes continued until 30 June 2021.

### 2.4. Sample Size Calculation

The sample size was calculated based on the primary outcome of all-cause mortality using Epi Info software (version 5.5.5). Prior registry data in Thai patients with heart failure reported a 10-year all-cause mortality of approximately 73.3% [18,19]. We hypothesized that influenza vaccination would provide at least a 13% relative reduction in this outcome, consistent with effect sizes observed in previous cohort studies and meta-analyses evaluating vaccination in high-risk cardiovascular populations [20].

A Chi-square test of two proportions with continuity correction was applied, assuming a 1:2 ratio of vaccinated to unvaccinated patients, 80% statistical power, and a 5% type I error rate. Based on these assumptions, the required minimum sample size was 422 patients (159 vaccinated and 318 unvaccinated). This calculation ensured adequate power to detect a clinically meaningful difference in mortality outcomes between groups, while also accounting for the smaller proportion of vaccinated individuals in the real-world Thai HF population.

### 2.5. Statistical Analysis

Normality of continuous variables was assessed using the Kolmogorov–Smirnov test. Patient characteristics were compared between vaccination groups using the Chi-square or Fisher’s exact test for categorical variables and the independent-sample *t*-test or Mann–Whitney U test for continuous variables, as appropriate.

Univariate and multivariable Cox proportional hazard regression models were used to assess the association between vaccination and the composite outcome of all-cause mortality or HF hospitalization. Vaccination status and all clinically relevant baseline covariates (including age, sex, comorbidities, LVEF, medications, and laboratory values) were included in multivariable models regardless of univariate significance. The proportional hazards assumption was evaluated using Schoenfeld residuals.

To account for baseline differences between groups, a propensity score model was constructed using logistic regression including all baseline covariates, and the area under the receiver operating characteristic curve (AUC) was reported to evaluate model performance. Inverse probability weighting with regression adjustment (IPWRA) was then applied to estimating the average treatment effect (ATE). The balance between groups was assessed using standardized mean differences (SMDs), with an SMD < 0.1 considered acceptable.

Because of potential survival bias in the vaccinated group, a subgroup analysis was conducted by stratifying vaccination within ≤1 year and >1 year after HF diagnosis.

All analyses were conducted using STATA version 16.0, and a two-sided *p* < 0.05 was considered statistically significant.

### 2.6. Role of Funding Source

This research received no specific grant from any funding agency in the public, commercial, or not-for-profit sectors.

## 3. Results

**Figure 1:**
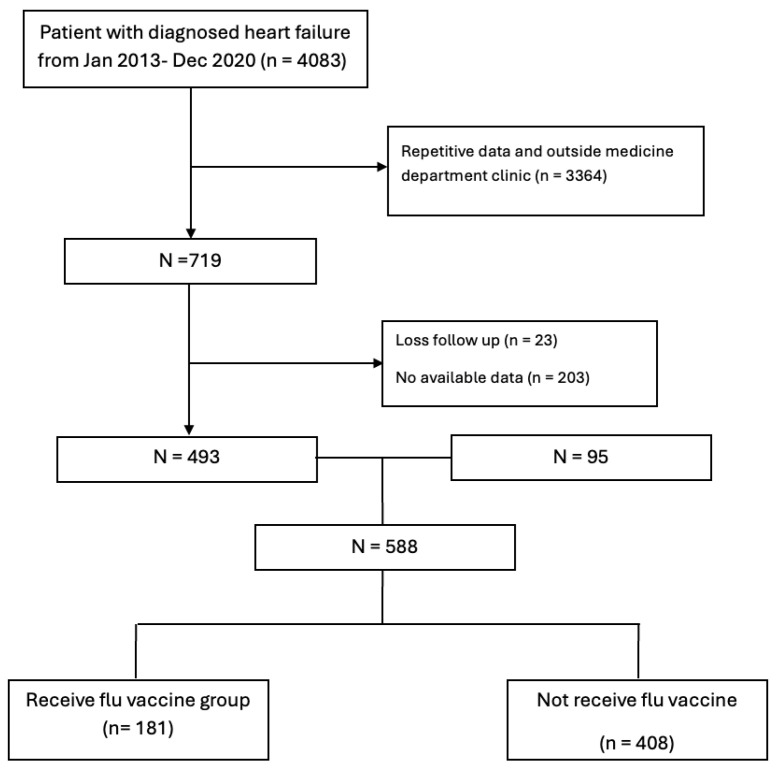
Study population in the study.

A total of 4083 patients with a diagnosis of HF between January 2013 and December 2020 were initially identified from hospital records. Of these, 3364 patients were excluded because of repetitive data or follow-up in non-medicine department clinics. Among the remaining 719 patients, 23 were excluded due to loss to follow-up for more than one year after the index diagnosis, and 203 were excluded because of unavailable data. The final analytic cohort therefore consisted of 588 patients, of whom 181 (30.8%) received at least one influenza vaccination during the follow-up period and 408 (69.2%) did not (Figure 1). The median follow-up time was 57 ± 27 months.

Baseline characteristics of the study population are summarized in Table 1. A total of 589 patients with heart failure were included, comprising 181 patients who received influenza vaccination and 408 who did not. Vaccinated patients were slightly younger (61.8 ± 16.1 vs. 67.0 ± 14.2 years, *p* < 0.001) and more often male (59.7% vs. 48.0%, *p* = 0.009). Distribution of health insurance schemes also differed, with vaccinated patients more frequently covered by government service/state enterprise programs (44.9% vs. 54.0%) and less frequently by universal coverage (30.7% vs. 24.7%, *p* = 0.001).

With respect to cardiovascular risk factors, vaccinated patients had lower rates of hypertension (50.3% vs. 65.7%, *p* < 0.001), diabetes mellitus (34.3% vs. 44.9%, *p* = 0.016), dyslipidemia (42.5% vs. 52.5%, *p* = 0.026), and chronic kidney disease (26.0% vs. 35.9%, *p* = 0.018). Body mass index was similar between groups (25.0 ± 6.1 vs. 25.0 ± 5.7 kg/m^2^, *p* = 0.98), as was diastolic blood pressure (72.6 ± 12.4 vs. 71.7 ± 11.0 mmHg, *p* = 0.43). However, vaccinated patients had a lower systolic blood pressure (119.5 ± 18.2 vs. 128.1 ± 21.8 mmHg, *p* < 0.001). The mean MAGGIC score was comparable between groups (19.0 ± 6.4 vs. 19.4 ± 6.5, *p* = 0.42).

Regarding HF subtype, vaccinated patients were more likely to have heart failure with a reduced ejection fraction (HFrEF) (63.5% vs. 32.4%, *p* < 0.001), whereas HF with a preserved ejection fraction (HFpEF) was more common in the unvaccinated group (53.7% vs. 25.4%, *p* < 0.001).

Medication use also differed between groups. Vaccinated patients were more likely to be treated with ACE inhibitors (37.6% vs. 25.5%, *p* = 0.003), ARNIs (7.2% vs. 2.0%, *p* = 0.002), beta-blockers (78.5% vs. 61.8%, *p* < 0.001), and MRAs (60.2% vs. 24.5%, *p* < 0.001). The use of ARBs, statins, and antiplatelet agents did not significantly differ between groups. The median number of guideline-directed medical therapy (GDMT) agents was higher in the vaccinated group (2.0 [IQR 1.0–2.0] vs. 1.0 [IQR 1.0–2.0], *p* < 0.001).

In a propensity model, significant predictors of influenza vaccination included the use of MRAs, HFmrEF/HFrEF status, number of GDMT medications, and DBP, whereas SBP was negatively associated (Table 2).

Univariate analysis was performed and identified influenza vaccination, age, gender, DBP, MAGGIC heart failure score, history of hypertension, diabetes, dyslipidemia, chronic kidney disease, treatments with ACEIs, MRAs, and antiplatelets, and number of GDMT medications as potential predictors of composite all-cause mortality or HF hospitalization (Table 3). A multivariate Cox proportional hazard model showed that influenza vaccination, gender, BMI, and a high MAGGIC HF score remained significant. After adjusting covariates, influenza vaccination was associated with a 56% lower risk of composite all-cause mortality or HF hospitalization (HR: 0.44; 95% CI: 0.31–0.63; *p* < 0.001) as shown in Table 4.

**Figure 2:**
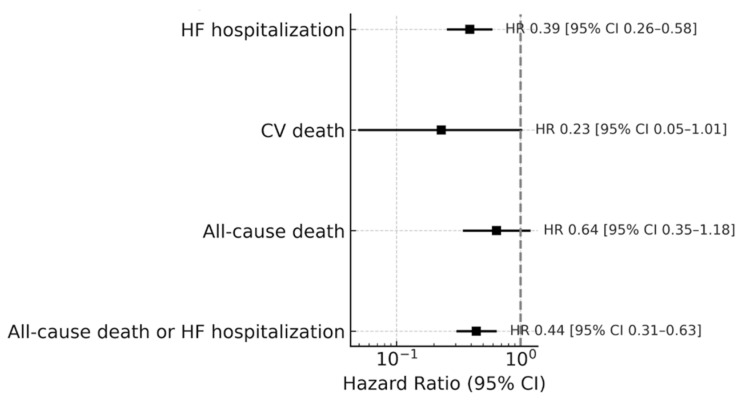
Adjusted effect sizes of influenza vaccination on composite of all-cause death or HF hospitalization and individual components.

Influenza vaccination could also significantly lower the risk of HF hospitalization (HR = 0.39; 95% CI: 0.26–0.58; *p* < 0.001), but the reductions in all-cause mortality (HR: 0.64; 95% CI: 0.35–1.18; *p* = 0.155) and CV death (HR: 0.23; 95% CI: 0.05–1.01; *p* = 0.052) were not statistically significant; see Figure 2.

The IPTW model was applied and achieved a good covariate balance; i.e., all weighted standardized mean differences were <0.10 (see Supplementary Document: Table S1). In the IPWRA model, the median time to the composite outcome was slightly shorter in the influenza vaccine group than that in the non-vaccine group, but this was not significant (ATE: −0.77 years; 95% CI: −2.56 to 1.04; *p* = 0.402). However, vaccination was associated with a longer median time to all-cause mortality [9.45 years (95% CI: 1.76–17.06); *p* = 0.016] and HF hospitalization [3.06 years (95% CI: 0.14–5.98; *p* = 0.04) than non-vaccination; see Table 5.

The median time to the first influenza vaccination was 13 months after an HF diagnosis; 65% received one dose, 23% received two doses, and 12% received more than two doses. A subgroup analysis showed that receiving an influenza vaccination within 1 year after HF diagnosis trended toward a higher risk for composite all-cause mortality or HF hospitalization (HR = 0.49 vs. 0.31), all-cause mortality (HR = 0.79 vs. 0.31), HF hospitalization (HR = 0.54 vs. 0.26), and CV death (HR = 0.30 vs. 0.13) compared to that in those receiving an influenza vaccination more than 1 year after an HF diagnosis; see Table 6.

## 4. Discussion

There are limited data on influenza vaccination rates in heart failure patients in Thailand. In patients with chronic heart failure, despite strong recommendations from the Heart Failure Council of Thailand advocating for annual vaccination, vaccination rates were only 31% in patients with heart failure even in this tertiary-care center. There were several reasons for poor influenza vaccine uptake in the heart failure population, such as the cost of vaccination and limited knowledge regarding the benefits of vaccination. This was a limited study investigating the association of an influenza vaccine and outcomes in heart failure patients in Thailand that had experienced an influenza infection for over a year (nonseasonal pattern). In this study, patients who received the influenza vaccine were more likely to be classified into the HFrEF group, which had a high mortality rate in the Thai ADHERE registry (HR: 1.47; 95% CI: 1.25–1.72) [20].

Our results differ from those of several large observational studies and meta-analyses that have reported significant reductions in both all-cause mortality and HF hospitalization with influenza vaccination. For example, Udell et al. demonstrated a 17% reduction in major adverse cardiovascular events in a meta-analysis of over 6000 patients [21,22], while Modin et al., using a Danish registry of more than 130,000 HF patients, showed that repeated annual vaccination was associated with progressively lower mortality [19]. The discrepancy of our study may reflect the shorter follow-up (median: 57 months vs. 10 years in prior studies), a lower observed event rate (38.5% vs. >70%), and potential residual confounding.

Recent randomized controlled trials have also provided mixed results. The IAMI trial (2021) demonstrated that influenza vaccination during hospitalization for acute myocardial infarction reduced the composite of all-cause mortality, myocardial infarction, or stent thrombosis, suggesting cardioprotective benefits in high-risk populations [23]. In contrast, the DANFLU-1 pilot trial (2022) in elderly patients with cardiovascular disease found no significant difference in outcomes, though it was underpowered for hard endpoints [24]. Importantly, a 2023 systematic review and meta-analysis specific to HF by Rodrigues et al. reinforced the protective effect of influenza vaccination on HF hospitalization and cardiovascular mortality [10]. Together, these findings suggest that while the magnitude of the benefit may vary, the overall direction of evidence favors vaccination as a preventive strategy in cardiovascular care.

Our study contributes novel data by examining influenza vaccination in a tropical, middle-income country where influenza circulates year-round rather than seasonally. This epidemiologic difference may influence vaccine effectiveness and underscores the importance of context-specific data. The consistent reduction in HF hospitalization observed in our cohort suggests that vaccination may alleviate one of the most burdensome aspects of HF care, with potential benefits for patients, caregivers, and healthcare systems.

From a policy perspective, these findings strengthen the rationale for integrating influenza vaccination into chronic HF management programs in Thailand. Targeted subsidies, inclusion in national HF registries, and linkage to community-based delivery systems could improve uptake and equity. As the burden of HF hospitalizations continues to grow, even a modest reduction could translate into substantial cost savings for health systems, particularly in resource-constrained settings.

In conclusion, while influenza vaccination was not associated with a statistically significant reduction in the composite of mortality and HF hospitalization in our study, the trend toward fewer HF hospitalizations is consistent with international evidence and supports vaccination as part of comprehensive HF care. Larger, adequately powered prospective studies in tropical Asian populations are needed to confirm these findings and guide policy on integrating influenza vaccination into guideline-directed management of HF.

### Limitations

Several limitations warrant consideration. First, the retrospective design is subject to missing or incomplete data, and despite physician review and supplemental verification, residual bias cannot be excluded. Second, unmeasured confounding remains possible, as vaccinated patients may have differed in their health behaviors or clinical engagement. Third, guarantee-time bias may have contributed to the observed differences between patients vaccinated within and after one year of HF diagnosis. Finally, the relatively modest sample size compared with large international registries may have limited the statistical power to detect differences in mortality outcomes.

Additionally, influenza vaccination data were derived exclusively from the hospital EMR system, and vaccinations administered outside the study hospitals could not be verified due to the absence of a national vaccination registry in Thailand. Vaccination records were verified through telephone interviews with patients or their caregivers to confirm accuracy; however, underreporting of prior influenza vaccination cannot be completely excluded due to the lack of a national registry. These factors may introduce potential information and selection bias. Low influenza vaccination coverage in Thailand (10–20% among high-risk groups) and limited vaccine accessibility may have contributed to the low prevalence of pre-diagnosis vaccination among the study population.

## 5. Conclusions

In patients with heart failure, influenza vaccination was not associated with a statistically significant reduction in the composite of all-cause death or HF hospitalization after adjustment for confounders. However, vaccination was consistently associated with a reduced risk of HF hospitalization, which is aligned with findings from international studies.

These results suggest that influenza vaccination may help alleviate one of the most burdensome aspects of HF care in Thailand, where influenza circulates year-round. From a healthcare policy perspective, strengthening vaccine uptake could reduce hospitalizations, improve patient outcomes, and lower healthcare costs. Integration of influenza vaccination into comprehensive HF management programs should therefore be encouraged.

Larger, prospective studies in tropical Asian populations are warranted to confirm these observations, address residual uncertainties, and inform future guideline recommendations.

## Figures and Tables

**Table 1:** Baseline characteristics by influenza vaccine use.

Characteristics	Flu Vaccine	No Flu Vaccine	*p* Value
	*n* = 181	*n* = 408	
Age, year, mean (SD)	61.8 (16.1)	67 (14.2)	<0.001
Male (%)	108 (59.7)	196 (48)	0.009
Health insurance, *n* (%)			
Universal coverage	54 (30.7)	96 (24.7)	0.001
Government service/state enterprise	79 (44.9)	210 (54)	
Social security service	19 (10.8)	14 (3.6)	
Self pay	24 (13.6)	69 (17.7)	
BMI, mean (SD)	25 (6.1)	25 (5.7)	0.98
SBP, mean (SD)	119.5 (18.2)	128.1 (21.8)	<0.001
DBP, mean (SD)	72.6 (12.4)	71.7 (11.0)	0.43
MAGGIC heart failure score, mean (SD)	19 (6.4)	19.4 (6.5)	0.42
Comorbidities			
Hypertension, *n* (%)	91 (50.3)	268 (65.7)	<0.001
Diabetes mellitus, *n* (%)	62 (34.3)	183 (44.9)	0.016
Dyslipidemia, *n* (%)	77 (42.5)	214 (52.5)	0.026
CKD, *n* (%)	47 (26)	146 (35.9)	0.018
Type of HF			
HFrEF, *n* (%) -Ejection fraction, (SD)	115 (63.5) 25.06 (7.26)	132 (32.4) 27.86 (6.95)	<0.001 0.002
HFmrEF, *n* (%) -Ejection fraction	20 (11) 44.8 (2.48)	132 (32.4) 42.88 (2.93)	0.33 0.011
HFpEF, *n* (%)	46 (25.4)	219 (53.7)	<0.001
Ischemic HF, *n* (%)	75 (41.4)	146 (35.8)	0.19
Medication			
ACEIs, *n* (%)	68 (37.6)	104 (25.5)	0.003
ARBs, *n* (%)	51 (28.2)	92 (22.5)	0.14
ARNIs, *n* (%)	13 (7.2)	8 (2)	0.002
Beta-blockers, *n* (%)	142 (78.5)	252 (61.8)	<0.001
MRAs, *n* (%)	109 (60.2)	100 (24.5)	<0.001
Antiplatelets, *n* (%)	83 (45.9)	220 (53.9)	0.071
Statins, *n* (%)	125 (69.1)	268 (65.7)	0.42
Number of GDMT medications, median (IQR)	2.0 (1.0, 2.0)	1.0 (1.0, 2.0)	<0.001

**Table 2:** Clinical predictors significantly associated with receiving influenza vaccine.

Predictors	OR (95% CI)	*p* Value
HFmrEF-HFrEF vs. HFpEF	1.72 (1.06–2.78)	0.028
MRAs	2.09 (1.19–3.68)	0.011
SBP	0.98 (0.96–0.99)	0.001
DBP	1.03 (1.01–1.05)	0.001
Number of GDMT medications	1.54 (1.12–2.11)	0.007

**Table 3:** Factors associated with all-cause mortality or HF hospitalization: univariate analysis.

Variables	HR	*p*	(95% CI)
Flu vaccine	0.42	<0.001	0.29–0.58
Age (year)	1.03	<0.001	1.02–1.04
Male	0.69	0.005	0.53–0.89
DBP	0.98	0.021	0.97–1.0
MAGGIC score	1.09	<0.001	1.06–1.11
Hypertension	1.54	0.003	1.16–2.04
Diabetic mellitus	1.58	0.001	1.22–2.05
Dyslipidemia	1.53	0.002	1.17–1.99
CKD	1.74	<0.001	1.34–2.28
ACEIs	0.72	0.035	0.53–0.98
MRAs	0.69	0.013	0.52–0.93
Antiplatelets	1.31	0.044	1.01–1.71
Number of GDMT medications	0.78	<0.001	0.69–0.89

All the variables from Table 1 were examined, and only those significant at *p* < 0.05 are shown in the univariate analysis. CI: confidence interval; HR: hazard ratio; DBP: diastolic blood pressure; CKD: chronic kidney disease; ACEI: angiotensin-converting enzyme inhibitor; MRA: mineralocorticoid receptor antagonist; GDMT: guideline-directed medical therapy.

**Table 4:** Multivariate analyses for predicting HF hospitalization or all-cause death.

Variables	*p*	HR	(95% CI)
Flu vaccine	<0.001	0.44	0.31–0.63
MAGGIC score	<0.001	1.10	1.07–1.13
Male	0.001	0.63	0.48–0.83
BMI	0.006	1.04	1.01–1.06

Multivariate analyses including all the variables in the univariate analysis. CI: confidence interval; HR: hazard ratio; HFrEF: heart failure with reduced ejection fraction, GDMT: guideline-directed medical therapy.

**Table 5:** Estimation of time to composite outcome and each component of composite outcome: a propensity score analysis by inverse probability weighting with regression adjustment model.

Outcomes	Median Time (Year)		Average Treatment Effect (ATE)	(95% CI)	*p* Value
	Flu Vaccine	No Flu Vaccine			
All-cause mortality or HF hospitalization	6.58	7.35	−0.77	−2.56–1.03	0.402
All-cause mortality	16.34	6.97	9.45	1.76–17.06	0.016
HF hospitalization	7.04	4.00	3.06	0.14–5.98	0.040
Cardiovascular mortality	5.62	5.82	−0.20	−5.5–0.40	0.90

**Table 6:** Sensitivity analyses for predicting HF hospitalization or all-cause death comparing between two subgroups divided by the duration of vaccination after HF diagnosis.

Outcomes	Incidence Rate/Year	HR (95% CI)	*p* Value
Composite all-cause death or HF hospitalization			
- No influenza vaccine	0.102	1	
- Received an influenza vaccine ≤ 12 mo	0.056	0.49 (0.29–0.84)	0.009
- Received an influenza vaccine > 12 mo	0.028	0.31 (0.17–0.55)	<0.001
All-cause death			
- No influenza vaccine	0.035	1	
- Received an influenza vaccine ≤ 12 mo	0.024	0.79 (0.38–1.64)	0.525
- Received an influenza vaccine > 12 mo	0.012	0.31 (0.14–0.69)	0.004
HF hospitalization			
- No influenza vaccine	0.1	1	
- Received an influenza vaccine ≤ 12 mo	0.056	0.54 (0.32–0.90)	0.018
- Received an influenza vaccine > 12 mo	0.028	0.26 (0.15–0.46)	0.001
Cardiovascular death			
- No influenza vaccine	0.01		
- Received an influenza vaccine ≤ 12 mo	0.003	0.30 (0.39–2.22)	0.237
- Received an influenza vaccine > 12 mo	0.002	0.13 (0.18–1.02)	0.052

## Data Availability

The data supporting the findings of this study are available from the corresponding author upon reasonable request.

## Supplementary Materials

The following supporting information can be downloaded at: https://www.mdpi.com/article/10.3390/vaccines13101055/s1, Table S1: Balance of factor associate with receive influenza vaccine.
