# Supplementary material for: Effect of Influenza Vaccination on Mortality and Heart Failure Hospitalization in Heart Failure Patients
Source: Vaccines (Basel). 2025 Oct 15;13(10):1055. doi: 10.3390/vaccines13101055 (PMC12567559; doi:10.3390/vaccines13101055)
Supplement: Supplementary file 1 [file vaccines-13-01055-s001.zip › vaccines-3873158-supplementary.pdf]

vaccines

Supplementary data

Table S1. Balance of factor associate with receive influenza vaccine

Estimation	Standardized differences		Variance ratio	
	Raw	Weighted	Raw	Weighted
HFpEF vs. none	-0.6572	0.0266	0.7234	1.0043
MRA vs. none	0.8131	-0.0253	1.2489	0.9842
Number of GDMT	0.8399	-0.0204	1.0296	1.1008
SBP	-0.4773	-0.0871	0.6486	0.6915
DBP	0.0688	0.0175	1.2721	0.9161

*Test for covariate balance p-value = 0.3696
